# Exogenous Antioxidants Improve the Accumulation of Saturated and Polyunsaturated Fatty Acids in *Schizochytrium* sp. PKU#Mn4

**DOI:** 10.3390/md19100559

**Published:** 2021-09-30

**Authors:** Sai Zhang, Xiaohong Chen, Biswarup Sen, Mohan Bai, Yaodong He, Guangyi Wang

**Affiliations:** 1 Center for Marine Environmental Ecology, School of Environmental Science and Engineering, Tianjin University, Tianjin 300072, China; zhangsai@tju.edu.cn (S.Z.); xh_chen2011@tju.edu.cn (X.C.); bsen@tju.edu.cn (B.S.); bmh@zju.edu.cn (M.B.); 2 Polar Research Institute of China, Shanghai 200136, China; 3 Key Laboratory of Systems Bioengineering (Ministry of Education), Tianjin University, Tianjin 300072, China

**Keywords:** thraustochytrids, antioxidants, saturated fatty acids, polyunsaturated fatty acids, reactive oxygen species, transcriptomics

## Abstract

Species of *Schizochytrium* are well known for their remarkable ability to produce lipids intracellularly. However, during their lipid accumulation, reactive oxygen species (ROS) are generated inevitably as byproducts, which if in excess results in lipid peroxidation. To alleviate such ROS-induced damage, seven different natural antioxidants (ascorbic acid, α-tocopherol, tea extract, melatonin, mannitol, sesamol, and butylated hydroxytoluene) were evaluated for their effects on the lipid accumulation in *Schizochytrium* sp. PKU#Mn4 using a fractional factorial design. Among the tested antioxidants, mannitol showed the best increment (44.98%) in total fatty acids concentration. However, the interaction effects of mannitol (1 g/L) and ascorbic acid (1 g/L) resulted in 2.26 ± 0.27 g/L and 1.45 ± 0.04 g/L of saturated and polyunsaturated fatty acids (SFA and PUFA), respectively, in batch fermentation. These concentrations were further increased to 7.68 ± 0.37 g/L (SFA) and 5.86 ± 0.03 g/L (PUFA) through fed-batch fermentation. Notably, the interaction effects yielded 103.7% and 49.6% increment in SFA and PUFA concentrations in batch fermentation. The possible mechanisms underlining those increments were an increased maximum growth rate of strain PKU#Mn4, alleviated ROS level, and the differential expression of lipid biosynthetic genes andupregulated catalase gene. This study provides an applicable strategy for improving the accumulation of SFA and PUFA in thraustochytrids by exogenous antioxidants and the underlying mechanisms.

## 1. Introduction

Thraustochytrids are marine unicellular heterotrophic protists that can naturally accumulate lipids up to 55% of their dry biomass [1] and are promising cell factories for high-value polyunsaturated fatty acids (PUFA), saturated fatty acids (SFA), and terpenoids [2]. The thraustochytrid strains belonging to genera *Aurantiochytrium*, *Schizochytrium*, and *Thraustochytrium* are particularly known for their extraordinary ability to produce lipids during glucose or glycerol fermentation [3,4,5,6]. Over the past two decades, several strategies towards enhancing the production of high-value fatty acids in oleaginous thraustochytrids were reported [7,8,9,10,11,12]. The environmental stressors inducing cellular stress in thraustochytrids, such as nitrogen starvation [13,14] and low temperature [15,16], are traditionally employed in improving the biomass and fatty acids content. However, the major limitation of most enhancement strategies is that they also induce intracellular accumulation of reactive oxygen species (ROS), which are reported to inhibit growth and lower the fatty acids content [17].

Considerable efforts were made in the past few decades to optimize the culture conditions for the efficient production of fatty acids by oleaginous microalgae [18]. Particularly, strategies based on ROS regulation were recently proposed to avoid the undesirable cell death and peroxidation of fatty acids induced by excessive ROS [18,19]. For example, the manipulation of the enzymatic antioxidant system through the overexpression of superoxide dismutase has shown up to a 38.5% decline in ROS level along with 32.9% higher PUFA production in the engineered strain of *Schizochytrium* sp. [20]. Similarly, an exogenous superoxide reductase gene expression in cyanobacteria also suppressed ROS accumulation and lipid peroxidation [21]. However, public health concerns over genetically modified strains limit the application of such strategies in the nutraceutical industry.

Approaches that enhance the intracellular antioxidant capacity seem promising for overcoming the ROS-induced adverse effects. One such approach towards the manipulation of antioxidant capacity includes the application of antioxidant supplementation [22]. Exogenous antioxidants such as ascorbic acid, phenolic compounds, etc., are reported as an important component of the ROS scavenging system [23]. These compounds could alleviate ROS levels by acting as an electron donor or by suppressing ROS generation through the chelation of transition metal ions [24]. Previous studies have shown promising results of exogenous antioxidants on the growth and lipid production in microalgae. For instance, the addition of melatonin lowered the ROS levels in *Monoraphidium* sp. QLY-1 and increased lipid accumulation by 1.22-fold [25]. The addition of sesamol increased the cell growth and PUFA synthesis in *Crypthecodinium cohnii* [26]. With the exogenous addition of ascorbic acid (9 g/L), the yield of docosahexaenoic acid (DHA) was increased by 30.4% in *Schizochytrium* sp. [27]. Similarly, the addition of flaxseed oil [28] or propyl gallate [29] to the culture medium of *Schizochytrium* sp. significantly improved the DHA yield or lipid accumulation, respectively. However, most of these studies focused on the evaluation of singular antioxidants and not on the interaction effects of mixed antioxidants. Moreover, the mechanisms reinforcing the elevated accumulation of lipids in thraustochytrids as a result of exogenous antioxidants addition remain poorly understood.

In this study, seven different inexpensive, natural antioxidants were screened for their effects on the cell growth and lipid production capacity of *Schizochytrium* sp. PKU# Mn4. The optimal levels of the best antioxidants and their interaction effects were determined and the possible mechanisms underlining the effects of antioxidant supplementation were also investigated. This study provides several lines of evidence that suggest that the supplementation of exogenous antioxidants is an effective strategy towards improving the accumulation of SFA and PUFA in thraustochytrids.

## 2. Results

### 2.1. Screening Potential Antioxidants

The ANOVA models for dry cell weight (DCW) and total fatty acids (TFA) yield were found to be significant (*p* < 0.05) in the 2-level fractional factorial design (FFD) screening experiment (Table 1 and Table 2). α-tocopherol and mannitol were significant model terms (*p* < 0.05) for DCW, while α-tocopherol, melatonin, and ascorbic acid interactions with mannitol, butylated hydroxytoluene (BHT), or sesamol were significant (*p* < 0.05) terms for TFA yield. The antioxidants, namely α-tocopherol, melatonin, and mannitol with significant effects on the DCW and TFA yield were selected for further concentration optimization experiments.

### 2.2. Effects of Different Concentrations of Single Antioxidants

**Figure 1:**
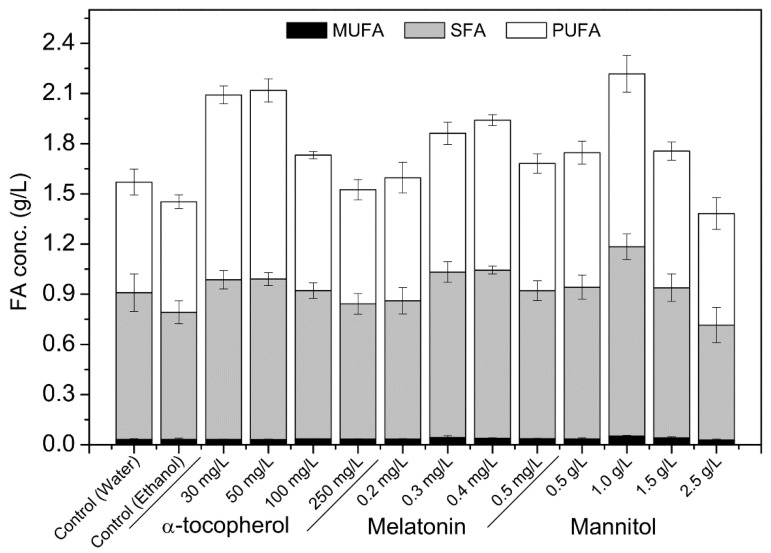
Effects of α-tocopherol, melatonin, and mannitol on the concentrations of different fatty acids accumulated by *Schizochytrium* PKU#Mn4 in batch culture. The data are expressed as mean ± SD of triplicate experiments.

The concentration optimization of α-tocopherol, melatonin, and mannitol revealed that these exogenous antioxidants show concentration-dependent effects. While these antioxidants showed little impact on DCW (Figure S1), their effects on the TFA accumulation were significant (Figure 1). The supplementation of α-tocopherol at 50 mg/L increased the TFA concentration by 45.85% (2.12 ± 0.10 g/L), and the PUFA and SFA concentrations by 70.85% (1.13 ± 0.07 g/L) and 26.21% (0.96 ± 0.04 g/L), respectively. With the optimal melatonin concentration (0.4 mg/L), the TFA concentration increased by 33.55% (1.94 ± 0.06 g/L), and the PUFA and SFA concentrations increased by 35.71% (0.90 ± 0.03 g/L) and 32.18% (1.0 ± 0.02 g/L), respectively. On the other hand, the optimal mannitol concentration (1.0 g/L) increased the TFA concentration by 44.98% (2.22 ± 0.19 g/L), and the PUFA and SFA concentrations by 56.31% (1.03 ± 0.11 g/L) and 28.96% (1.13 ± 0.08 g/L), respectively. Of these three tested antioxidants, the supplementation of mannitol (1.0 g/L) yielded the best TFA concentration.

### 2.3. Interaction Effects of Ascorbic Acid and Mannitol in Batch Culture

The DCW and TFA concentration declined with the increasing concentration of ascorbic acid in the mannitol and ascorbic acid supplement (Table 3). With the mannitol (1 g/L) and ascorbic acid (1 g/L) supplementation (MA), the DCW and TFA concentration increased by up to 54.1% (8.49 ± 0.18 g/L) and 78.2% (3.78 ± 0.26 g/L), respectively. The TFA produced with MA constituted 2.26 ± 0.27 g/L SFA, 1.45 ± 0.04 g/L PUFA, and 0.08 ± 0.01 g/L MUFA, which increased by 103.7%, 49.6%, and 82.0%, respectively. Furthermore, the addition of ascorbic acid at the concentration range of 1 to 6 g/L showed a notable rise in the SFA/PUFA ratio when compared with that without supplementation.

**Figure 2:**
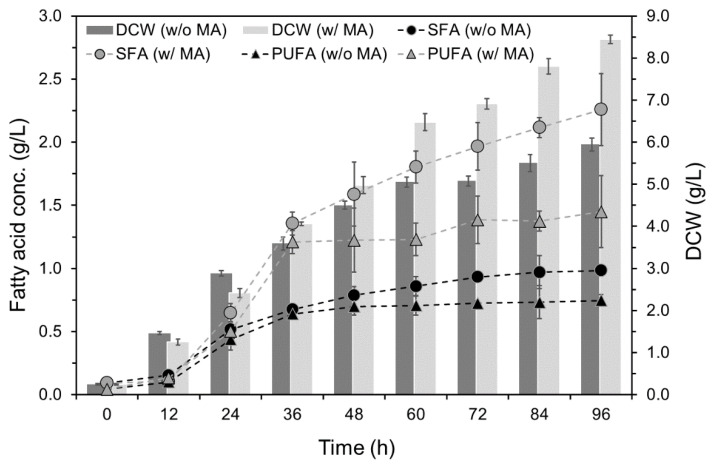
Time course of dry cell weight and concentrations of fatty acids accumulated by *Schizochytrium* PKU#Mn4 in batch culture supplemented with mannitol (1 g/L) and ascorbic acid (1 g/L) mixture. ‘w/o MA’ and ‘w/MA’ stand for without and with mannitol (1 g/L) and ascorbic acid (1 g/L) supplementation. The data are expressed as mean ± SD of triplicate experiments.

The time course of DCW, SFA, and PUFA concentrations upon MA supplementation exhibited sigmoid patterns (Figure 2), and the modified Gompertz model fitted these data well (Figure S2). The model predicted a maximum growth rate of 0.118 g/L∙h^−1^ for the culture with the supplement, which was 15.7% higher than that of the culture without the supplement (Table 4). Similarly, the model also predicted the maximum accumulation rates of SFA and PUFA to be 0.047 g/L∙h^−1^ and 0.056 g/L∙h^−1^, respectively, which were 123.8% and 86.7% higher than that of without supplementation. Both experimental (Figure 2) and predicted (Figure S2) data revealed a marked increase in DCW, SFA, and PUFA concentrations of the culture with supplementation compared with that of without supplementation from 36 h until the end of fermentation.

**Figure 3:**
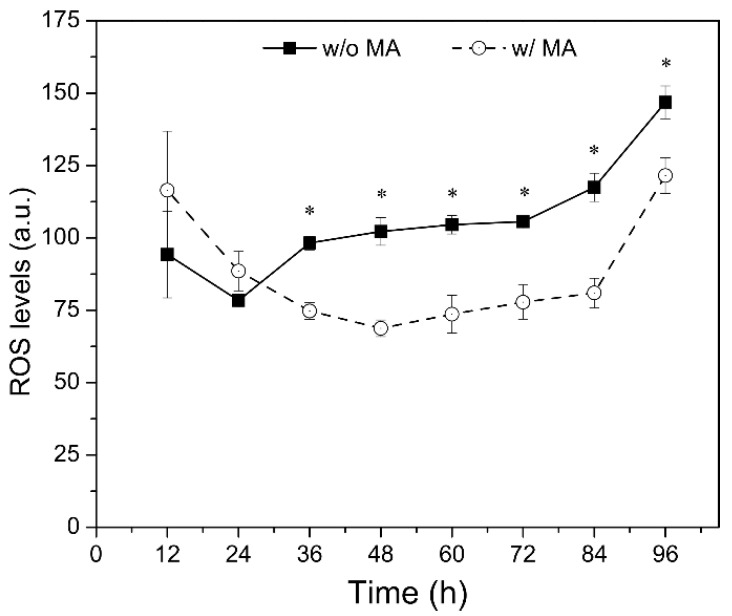
Time course of intracellular ROS levels in the batch culture of *Schizochytrium* PKU# Mn4 supplemented with mannitol (1 g/L) and ascorbic acid (1 g/L) mixture. * indicates the data have statistical significance at *p* < 0.05. ‘w/o MA’ and ‘w/MA’ stand for without and with mannitol (1 g/L) and ascorbic acid (1 g/L) supplementation. The data are expressed as mean ± SD of triplicate experiments.

The time course of intracellular ROS level in the batch culture with and without MA supplementation exhibited somewhat different patterns (Figure 3). Without the supplement, the ROS level declined until the first 24 h but increased thereafter until the end of fermentation. Whereas, with the supplement, the ROS level decreased until 48 h and then increased gradually until the end of fermentation. Notably, the ROS levels were significantly (*p* < 0.05) lower in the culture with MA supplementation from 36 h until the end of fermentation.

### 2.4. Effects of Mannitol and Ascorbic Acid Supplemention on Fed-Batch Culture

**Figure 4:**
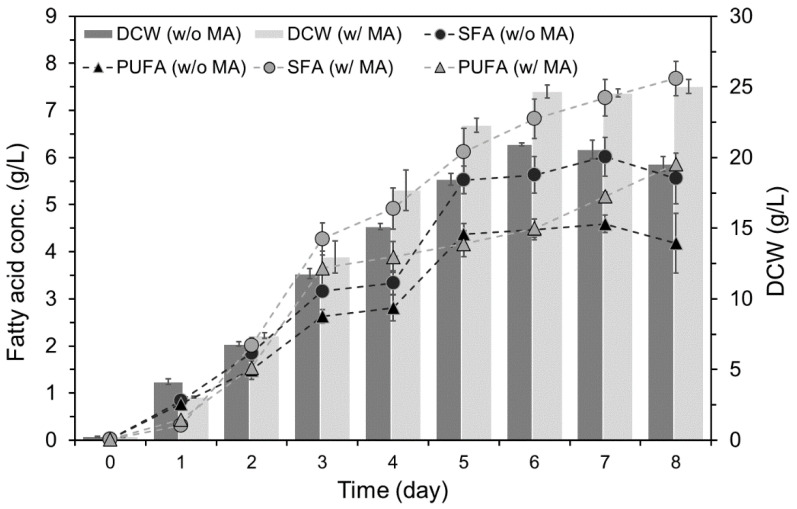
Time course of dry cell weight and concentrations of fatty acids accumulated by *Schizochytrium* PKU#Mn4 in fed-batch culture (5 L) supplemented with the antioxidant mixture (MA). ‘w/o MA’ and ‘w/MA’ stand for without and with mannitol (1 g/L) and ascorbic acid (1 g/L) supplementation. The data are expressed as mean ± SD of triplicate experiments.

In the fed-batch fermentation, the accumulation of the fatty acid with supplementation increased until the end of fermentation (i.e., day 8), while their accumulation without supplementation increased only until day 6 of fermentation (Figure 4). The maximum growth with or without MA was achieved at the same time (i.e., day 6); however, the growth with MA remained significantly higher from day 4 until the end of the fermentation. The maximum DCW achieved with MA in fed-batch fermentation was 25.03 ± 0.51 g/L, an increase of 19.71%. The accumulation patterns of SFA and PUFA were strongly (*p* < 0.001) correlated with the growth patterns during fermentation with and without MA. However, the accumulation of these fatty acids was generally better with MA. The MA supplementation yielded the maximum SFA and PUFA concentrations of 7.68 ± 0.37 g/L and 5.86 ± 0.03 g/L, which were 38.1% and 40.2% greater than that of the culture without MA supplementation.

### 2.5. Effects of Mannitol and Ascorbic Acid Supplementation at the Transcriptome Level

The transcriptome-level changes were analyzed for the cells harvested from the culture samples collected at 48 h of batch fermentation. A total of 33,813,639 (with MA group) and 21,621,092 (without MA group) clean reads were obtained, and over 94.42% and 93.42% of these reads were mapped to the reference genome. Further analyses identified 1210 differentially expressed genes (DEGs), of which 577 and 633 were upregulated and downregulated in the group with MA supplementation, respectively. The DEGs were further annotated against the GO database for functional information, which revealed that 39.79%, 36.81%, and 23.4% of the DEGs belonged to the biological process category, cellular component, and molecular function, respectively (Figure S3).

**Figure 5:**
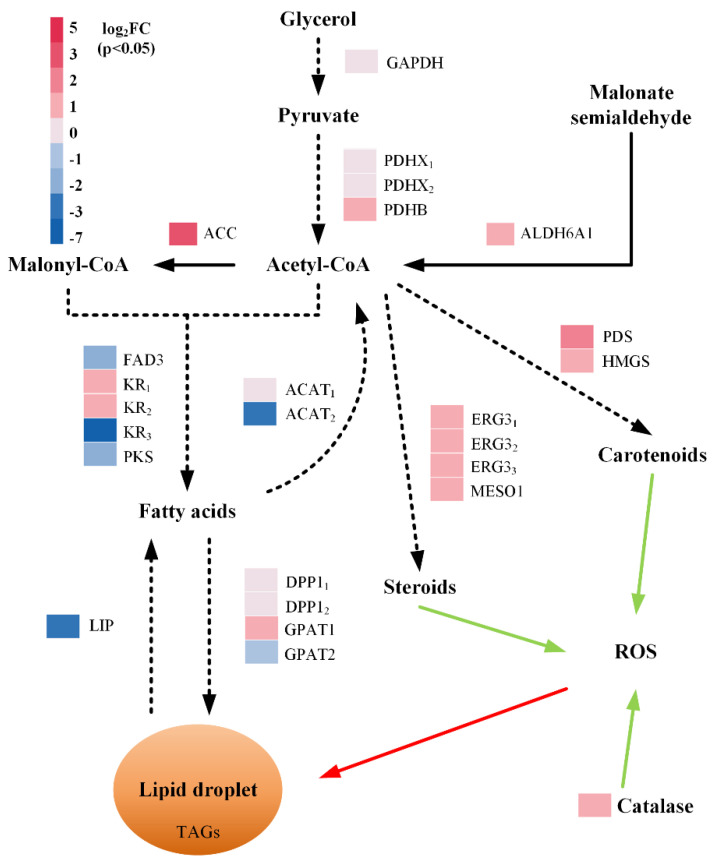
Differentially abundant genes and transcripts involved in lipid metabolism and ROS detoxification upon supplementation of mannitol (1 g/L) and ascorbic acid (1 g/L) mixture in batch fermentation. The solid and dotted black arrows indicate single-step and multiple-step pathways, respectively. The red solid arrow indicates the route of ROS-induced lipid peroxidation and the green solid arrow indicates the routes for reduction in ROS. GAPDH, glyceraldehyde-3-phosphate dehydrogenase; PDHX, pyruvate dehydrogenase complex; PDHB, pyruvate dehydrogenase E1 component subunit beta; ALDH6A1, malonate-semialdehyde dehydrogenase; ACC, acetyl-CoA carboxylase; FAD3, ω-3 desaturase; KR, ketoacyl-reductase; PKS, polyketide synthase; ACAT, acetyl-CoA acetyltransferase; DPP1, diacylglycerol pyrophosphate phosphatase 1; GPAT1, glycerol-3-phosphate O-acyltransferase 1; GPAT2, glycerol-3-phosphate O-acyltransferase 2; LIP, triacylglycerol lipase; ERG3, delta(7)-sterol 5(6)-desaturase; MESO1, methylsterol monooxygenase; PDS, phytoene desaturase; HMGS, hydroxymethylglutaryl-CoA synthase; CAT, catalase.

Several DEGs and transcripts related to the generation of acetyl-CoA and malonyl-CoA were upregulated, including genes encoding glyceraldehyde-3-phosphate dehydrogenase (GAPDH), pyruvate dehydrogenase transcripts (PDHX1, PDHX2, and PDHB), malonate-semialdehyde dehydrogenase (ALDH6A1), and acetyl-CoA carboxylase (ACC) (Figure 5). Two transcripts of the ketoacyl-reductase (KR) gene involved in the biosynthesis of fatty acids from the pool of acetyl-CoA and malonyl-CoA were also upregulated. However, one transcript of the KR gene and two genes encoding ω-3 desaturase (FAD3) and polyketide synthase (PKS) were downregulated at the same time. Furthermore, two transcripts encoding diacylglycerol pyrophosphate phosphatase 1 (DPP1_1_ and DPP1_2_) and one transcript encoding glycerol-3-phosphate O-acyltransferase 1 (GPAT1) involved in the process of lipid biosynthesis were upregulated. While the gene encoding triacylglycerol lipase (LIP) was downregulated concurrently.

In addition to the genes and transcripts involved in the lipid biosynthetic pathway, certain genes and transcripts encoding enzymes that catalyze the biosynthesis of steroids and carotenoids were also differentially expressed. Those included three transcripts of gene encoding delta(7)-sterol 5(6)-desaturase (ERG3_1_, ERG3_2_, and ERG3_3_), and genes encoding methylsterol monooxygenase 1 (MSMO1), phytoene desaturase (PDS), and hydroxymethylglutaryl-CoA synthase (HMGS), which were all upregulated. Furthermore, the gene encoding the enzyme catalase (CAT), which neutralizes hydrogen peroxide, was upregulated.

## 3. Discussion

### 3.1. Antioxidants Affect Growth and Fatty Acids Accumulation

The supplementation of antioxidants during fermentation were one of the most effective strategies to control the intracellular ROS levels and improve the production of fatty acids for several microbial strains [22]. In this study, the effects of seven different antioxidants and their interactions on the growth and fatty acid production were first evaluated and then the optimal concentrations of the potential antioxidants were determined. The most potent antioxidants, namely α-tocopherol, mannitol, and melatonin, at their optimal concentrations could significantly improve the production of fatty acids (Figure 1). However, each of these antioxidants yielded different effects on the accumulation of SFA and PUFA. For example, the supplementation of α-tocopherol and mannitol increased the accumulation of PUFA more than SFA, while the melatonin addition uniformly raised the accumulation of SFA and PUFA. Such differential effects of antioxidants can be useful while manipulating the selective fatty acid production for application in the biodiesel or nutraceuticals industry.

This study revealed that not all seven antioxidants are effective in improving the accumulation of the fatty acids in the strain PKU#Mn4. For instance, BHT was reported to enhance the lipid content of *Haematococcus pluvialis* LUGU and stimulate lipid accumulation in *Schizochytrium* sp. S31 [29,30]. However, in this study, the BHT supplementation did not show a promising effect on the lipid production of strain PKU#Mn4. Similarly, ascorbic acid, which was previously reported to improve the yield of certain PUFA in *Schizochytrium* sp. HX-308 by 30.44% [27], did show any significant effects on the accumulation of fatty acids in strain PKU#Mn4. Nevertheless, our results showed that melatonin, which was previously investigated for its application in improving fatty acid productivity in autotrophic microalgae [31,32,33], could markedly increase the accumulation of SFA and PUFA in thraustochytrids.

Based on the ANOVA results of the 2-level FFD experiment (Table 1), we evaluated the potential interaction effects of mannitol and ascorbic acid mixture by varying the concentration of ascorbic acid in the mixture. Our results suggested that the interaction between these two antioxidants could significantly promote the accumulation of SFA and PUFA under both batch and fed-batch fermentation conditions. Furthermore, it was realized that alleviation of ROS level during fermentation (Figure 3), and increased maximum growth rate of strain PKU#Mn4 (Table 4) were probable physiological mechanisms underlining the increased growth and accumulation of SFA and PUFA with MA supplement.

### 3.2. Exogenous Antioxidants Trigger Differential Expression of Lipid Biosynthetic Genes

The possible molecular mechanisms supporting the increased accumulation of fatty acids with MA supplementation were inferred by identifying the DEGs involved in lipid metabolism. Several DEGs were identified which catalyze reactions in the acetyl-CoA, fatty acids, lipids, steroids, and carotenoid biosynthetic pathways. One of those DEGs, encoding acetyl-CoA carboxylase, catalyzes the conversion of acetyl-CoA to malonyl-CoA—the rate-limiting step of fatty acid biosynthesis [34]. The upregulation of this gene and concomitant increase of fatty acids observed in the present study was in close agreement with the previous studies which have shown that increased expression of *acc* gene increases the precursor pool and promotes fatty acid biosynthesis [35,36,37]. Furthermore, the downregulation of the gene encoding enzyme LIP, which catalyzes the hydrolysis of triacylglycerols, suggested that antioxidant supplement may also suppress lipid degradation in strain PKU#Mn4. Overall, our study provides evidence that the MA supplementation can upregulate the build-up of the acetyl-CoA pool, and simultaneously suppress the oxidation of fatty acids, thereby increasing the accumulation of SFA and PUFA in thraustochytrids.

The importance of exogenous antioxidants supplementation in cellular ROS scavenging is well established [23]. However, it is not clear through which mechanisms the exogenous antioxidants alleviate cellular ROS levels. Previous studies have reported that exogenous melatonin alleviates the ROS level, suppresses lipid peroxidation, and elevates the activity of antioxidant enzymes in *Monoraphidium* sp. QLY-1 [25]. The addition of fulvic acid and EDTA during the cultivation of *Schizochytrium* sp. HX-308 showed a 44.7% and 81.9% increase in the activities of superoxide dismutase and catalase [19]. Along similar lines, our study revealed that the addition of exogenous antioxidants leads to the upregulation of catalase in strain PKU#Mn4, which is one of the key antioxidant enzymes that mediates intracellular ROS scavenging.

In conclusion, the interaction effects of 1 g/L each of mannitol and ascorbic acid could significantly augment growth, SFA, and PUFA accumulation, and also alleviate the ROS level during fermentation. More importantly, the findings of this study significantly advance the knowledge about the mechanisms that underpin the upscaled accumulation of lipids in thraustochytrids under the influence of exogenous antioxidants.

## 4. Materials and Methods

### 4.1. Microorganism and Batch Fermentation

*Schizochytrium* sp. PKU#Mn4, previously isolated from the Pearl River Delta region of China [38], was used in this study. The strain was maintained on 1% modified Vishniac’s (MV) agar plates at room temperature and subcultured every 4 weeks [39].

Seed culture medium and conditions were used as described in our previous study [20]. A 5% (*v/v*) seed culture was transferred to a 100 mL shake flask containing 40 mL of fermentation medium and cultured at 28 °C with a constant orbital shaking speed of 170 rpm for 4 days. The fermentation medium contained 40 g/L glycerol, 2.5 g/L yeast extract, 0.25 g/L KH_2_PO_4_, and 33 g/L sea salt [40]. A 10× stock solution of each antioxidant was prepared by dissolving in water or ethanol (EtOH) based on its solubility and then filtered before addition into the medium. Ascorbic acid, mannitol, and tea extracts were dissolved in water, while α-tocopherol, melatonin, sesamol, and BHT were dissolved in ethanol. A control flask was prepared to contain an equal amount of water or ethanol.

### 4.2. Experimental Design and Statistical Analyses

Seven different natural antioxidants, namely ascorbic acid, α-tocopherol, tea extract, melatonin, mannitol, sesamol, and BHT were screened based on a two-level FFD (Table 1). According to the FFD, the total number of experimental runs was 16 (Table S1), each carried out in triplicate. The antioxidant compounds were added to the fermentation culture and not to the seed culture. The response variables, DCW (g/L), and TFA yield (mg/g DCW), for each run, were measured at 96 h of batch cultivation. Design-Expert software (version 10.0.7, Stat-Ease Co. Inc., Minneapolis, MN, USA) was used for the experimental design and regression analysis of the experimental data. A modified Gompertz model [41] was fitted to the experimental data to estimate the parameters *a* (maximum growth potential, g/L), *R* (maximum growth rate, g/L·h^−1^), and *λ* (lag time, h) using R software (version 4.0.0, https://www.r-project.org, accessed on 8 May 2019).

### 4.3. Quantification of Dry Cell Weight and Fatty Acids

The cells in the culture broth were collected, lyophilized, and their dry weight was determined following the procedures described in our previous study [20]. Using the lyophilized cells (30 mg), a one-step transesterification process was performed to prepare the fatty acids methyl esters (FAMEs) [20], which were then resolved on a gas chromatography 7890B (Agilent Technologies, Santa Clara, CA, USA) system equipped with DB-23 capillary column (60 m × 0.25 μm × 0.32 μm) (Agilent Technologies, Santa Clara, CA, USA) and nitrogen as the carrier gas. The FAMEs were quantified by comparing the retention time of each peak with the one in 37 Component FAME Mix Standard (Supelco Co. Inc., Bellefonte, PA, USA). C16:0 was the major SFA constituent, C22:6 and C22:5 were the major PUFA constituents, and C14:1, C15:1, and C17:1 were the MUFA constituents.

### 4.4. Determination of Intracellular ROS

The intracellular ROS levels were measured with ROS Assay Kit (Beyotime Biotechnology, Shanghai, China) following the manufacturer’s instructions. In brief, 1 × 10^6^ to 2 × 10^7^ cells were harvested by centrifugation at 4000 rpm for 10 min, and the pellet was then washed and re-suspended in 1 mL artificial seawater. One μL of 2′-7′-dichlorofluorescin diacetate (DCFH-DA) was added to the cell suspension and incubated at 37 °C for 20 min. After DCFH-DA treatment, the cell suspension was washed thrice with artificial seawater to remove the excess DCFH-DA. The fluorescence intensity was detected with a fluorescence spectrophotometer (F97 Pro, Lengguang Technology Co. Inc., Shanghai, China) at the excitation and emission wavelengths of 488 nm and 525 nm, respectively.

### 4.5. Fed-Batch Fermentation

Fed-batch fermentation was performed in a 5-L bioreactor (Shanghai Dong Ming Industrial Co. Ltd., Shanghai, China) at 28 °C for 8 days as described in our previous study [40]. In brief, 300 mL seed culture was prepared as described in Section 4.1 and inoculated into a 2 L fermentation medium with or without antioxidant mixture (1 g/L each of mannitol and ascorbic acid). The agitation rate of the bioreactor was altered to maintain the dissolved oxygen (DO) at 50% from day 0 to day 2 and at 10% for the rest of the fermentation period. On day 2 and day 3, a 100 mL feed medium (400 g/L glycerol and 25 g/L yeast extract) was added to the fermentation broth. An aliquot of the culture was harvested every 24 h to determine the DCW and fatty acids concentration following the methods described in Section 2.3.

### 4.6. Transcriptome Analysis

The changes in the transcriptome of strain PKU#Mn4 under the influence of antioxidants (1 g/L each of mannitol and ascorbic acid) supplementation in the batch culture were investigated by the RNA-seq method. Three parallel samples (10 mL) of culture broth at 48 h of fermentation, without (control group) or with (test group) antioxidants, were collected and the cells were harvested, frozen with liquid nitrogen, and stored at −80 °C. Total RNA extraction, its quality, integrity, and quantity checks were conducted by BioMarker Technologies (Beijing, China). For each sample, 1 μg RNA was used to generate the library with the NEBNext Ultra TM RNA Library Prep Kit for Illumina (New England BioLabs Inc., Ipswich, MA, USA). The clustering of the index-coded samples was carried out on a cBot Cluster Generation System using TruSeq PE Cluster Kit v4-cBot-HS (Illumina Inc., San Diego, CA, USA) according to the manufacturer’s instructions. The library was sequenced on an Illumina platform and paired-end reads were generated. All analyses were based on clean data with high-quality reads after removing those that contained adapter, poly-N, or low-quality reads from the raw data. The clean reads were first mapped to the reference genome with HISAT2 [42] and then assembled with StringTie [43]. The gene expression levels were quantified by the FPKM method [44], and the differential expression analysis was carried out with DEGseq [45]. The genes or transcripts with a false discovery rate (FDR) less than 0.05 and the absolute value of log_2_ (fold change (FC)) > 1 were regarded as the differentially expressed genes (DEGs). RNA sequencing was performed by BioMarker Technologies (Beijing, China), and the sequencing data analysis was done by BMKCloud (www.biocloud.net, accessed on 8 May 2019).

## Figures and Tables

**Table 1:** Low- and high-level concentrations of individual antioxidants.

Factor	Antioxidant	Low Level (−1)	High Level (+1)
A	Ascorbic acid	9.0 g/L	13.5 g/L
B	α-tocopherol	0.50 g/L	0.75 g/L
C	Tea extract	0.50 g/L	0.75 g/L
D	Melatonin	0.25 mg/L	0.375 mg/L
E	Mannitol	1.0 g/L	1.5 g/L
F	Sesamol	70 mg/L	105 mg/L
G	BHT	2 mg/L	3 mg/L

**Table 2:** Significance of ANOVA model terms for the response variables DCW and TFA yield.

Source	*p*-Value	Source	*p*-Value
Response 1: DCW (g/L)		Response 2: TFA (mg/g DCW)	
Model	0.0447	Model	0.0180
A: ascorbic acid	0.3917	A: ascorbic acid	0.6802
B: α-tocopherol	0.0406	B: α-tocopherol	0.0203
C: tea extract	0.1094	D: melatonin	0.0206
D: melatonin	0.1397	E: mannitol	0.1194
E: mannitol	0.0141	F: sesamol	0.1643
F: sesamol	0.4890	G: BHT	0.6473
AE	0.1069	AE	0.0487
AF	0.1061	AF	0.0166
		AG	0.0047
		BD	0.0697

**Table 3:** Interaction effects of various combinations of antioxidants on the biomass and fatty acids production in batch culture of *Schizochytrium* PKU#Mn4.

Treatment	DCW (g/L)	MUFA (g/L)	SFA (g/L)	PUFA (g/L)	SFA/PUFA
Control	5.89 ± 0.23	0.03 ± 0.00	0.88 ± 0.11	0.66 ± 0.08	1.32
Mannitol (1 g/L)	5.51 ± 0.33	0.04 ± 0.01	1.11 ± 0.17	0.97 ± 0.13	1.14
Mannitol (1 g/L) + Ascorbic acid (1 g/L)	8.49 ± 0.18	0.08 ± 0.01	2.26 ± 0.27	1.45 ± 0.04	1.56
Mannitol (1 g/L) + Ascorbic acid (2 g/L)	7.96 ± 0.28	0.06 ± 0.01	2.26 ± 0.07	1.19 ± 0.09	1.89
Mannitol (1 g/L) + Ascorbic acid (3 g/L)	6.79 ± 0.12	0.04 ± 0.00	1.87 ± 0.16	1.01 ± 0.14	1.85
Mannitol (1 g/L) + Ascorbic acid (6 g/L)	4.67 ± 0.33	0.04 ± 0.00	1.31 ± 0.19	0.73 ± 0.07	1.80
Mannitol (1 g/L) + Ascorbic acid (9 g/L)	3.38 ± 0.00	0.02 ± 0.00	0.79 ± 0.06	0.59 ± 0.02	1.35
Mannitol (1 g/L) + Ascorbic acid (12 g/L)	1.84 ± 0.03	0.01 ± 0.00	0.45 ± 0.02	0.34 ± 0.03	1.32
Mannitol (1 g/L) + Ascorbic acid (15 g/L)	1.22 ± 0.02	0.01 ± 0.00	0.29 ± 0.02	0.28 ± 0.03	1.07

Note: The results are expressed as mean ± SD of triplicate experiments.

**Table 4:** Estimates of modified Gompertz model parameters after fitting experimental data.

Dependent Variable		*a* (g/L)	*R* (g/L·h^−1^)	λ (h)	Residual Standard Error
DCW	w/o MA	5.799 ***	0.102 ***	−1.791	0.2223
	w/MA	9.276 ***	0.118 ***	3.478	0.2045
SFA	w/o MA	0.977 ***	0.021 ***	1.763	0.0434
	w/MA	2.191 ***	0.047 ***	9.832 *	0.1017
PUFA	w/o MA	0.729 ***	0.030 ***	9.069 ***	0.0194
	w/MA	1.369 ***	0.056 **	13.723 **	0.0948

Note: ‘w/o MA’ and ‘w/MA’ stand for without and with mannitol (1 g/L) and ascorbic acid (1 g/L) supplementation; significance codes: *** 0.001, ** 0.01, * 0.05.

## Data Availability

Not applicable.

## Supplementary Materials

The following are available online at https://www.mdpi.com/article/10.3390/md19100559/s1, Figure S1: Effects of α-tocopherol, melatonin, and mannitol on the dry cell weight (DCW) of *Schizochytrium* PKU#Mn4 culture; Figure S2: Experimental data and prediction using modified Gompertz model; Figure S3: GO classification of differentially expressed genes; Table S1: Experiment design and response values for screening antioxidants.
