# Supplementary material for: Exogenous Antioxidants Improve the Accumulation of Saturated and Polyunsaturated Fatty Acids in Schizochytrium sp. PKU#Mn4
Source: Mar Drugs. 2021 Sep 30;19(10):559. doi: 10.3390/md19100559 (PMC8541261; doi:10.3390/md19100559)
Supplement: Supplementary file 1 [file marinedrugs-19-00559-s001.zip › marinedrugs-1387732-supplementary.pdf]

Supplementary information

Exogenous Antioxidants Improve the Accumulation of Saturated and Polyunsaturated Fatty Acids in *Schizochytrium* sp. PKU#Mn4

Sai Zhang[1,2,†], Xiaohong Chen[1,†], Biswarup Sen[1], Mohan Bai[1], Yaodong He[1,*], Guangyi Wang[1, 3,*]

[1]Center for Marine Environmental Ecology, School of Environmental Science and Engineering, Tianjin University, Tianjin 300072, China

[2]Polar Research Institute of China, Shanghai 200136, China

[3]Key Laboratory of Systems Bioengineering (Ministry of Education), Tianjin University, Tianjin 300072, China

***Corresponding author.**

E-mail: yaodong.he@tju.edu.cn; gywang@tju.edu.cn

Table S1. Experiment design and response values for screening antioxidants.

Run	A	B	C	D	E	F	G	Response DCW (g/L)	Response TFA (mg/g DCW)
1	1	-1	1	1	-1	-1	1	5.37	388.91
2	-1	-1	-1	1	-1	1	1	4.82	275.76
3	-1	-1	1	1	1	-1	-1	5.06	413.74
4	1	-1	-1	1	1	1	-1	5.07	346.80
5	1	1	-1	1	-1	-1	-1	4.82	328.25
6	-1	1	-1	1	1	-1	1	4.81	406.51
7	1	-1	1	-1	-1	1	-1	5.66	278.90
8	1	1	-1	-1	-1	1	1	5.24	386.31
9	1	1	1	1	1	1	1	4.57	395.81
10	-1	1	1	-1	-1	-1	1	5.22	324.15
11	-1	-1	-1	-1	-1	-1	-1	5.54	351.40
12	-1	1	1	1	-1	1	-1	5.10	339.30
13	1	-1	-1	-1	1	-1	1	4.49	289.09
14	-1	1	-1	-1	1	1	-1	4.76	395.02
15	1	1	1	-1	1	-1	-1	4.73	320.20
16	-1	-1	1	-1	1	1	1	5.45	265.63

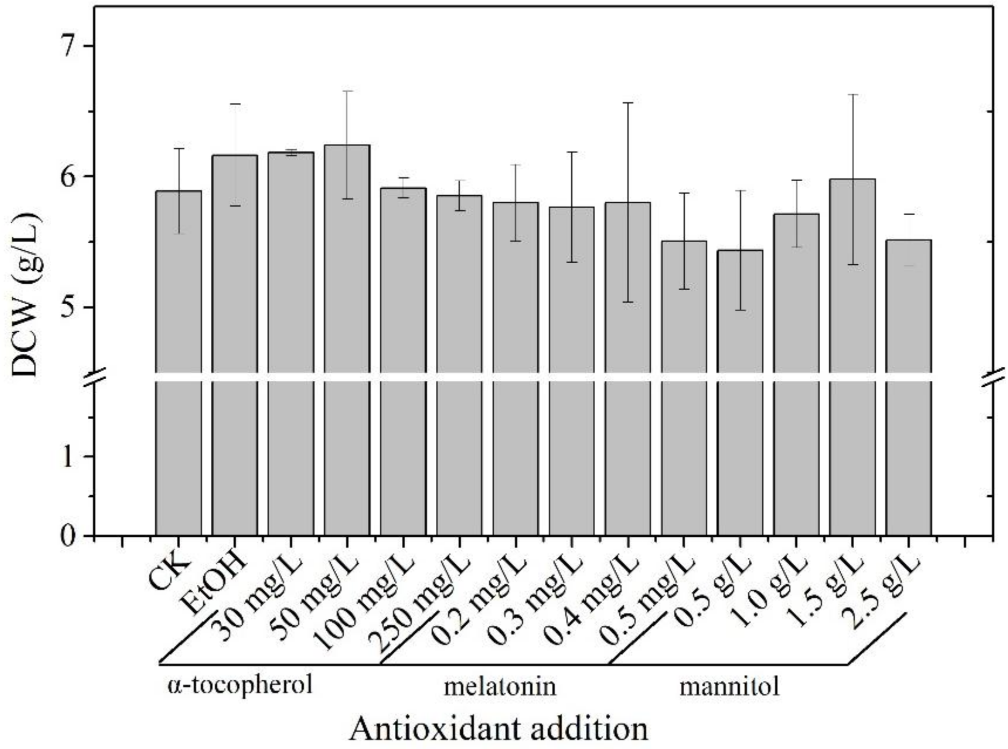

Figure S1. Effects of α-tocopherol, melatonin, and mannitol on the dry cell weight (DCW) of *Schizochytrium* PKU#Mn4 culture. CK stands for the control group with water.

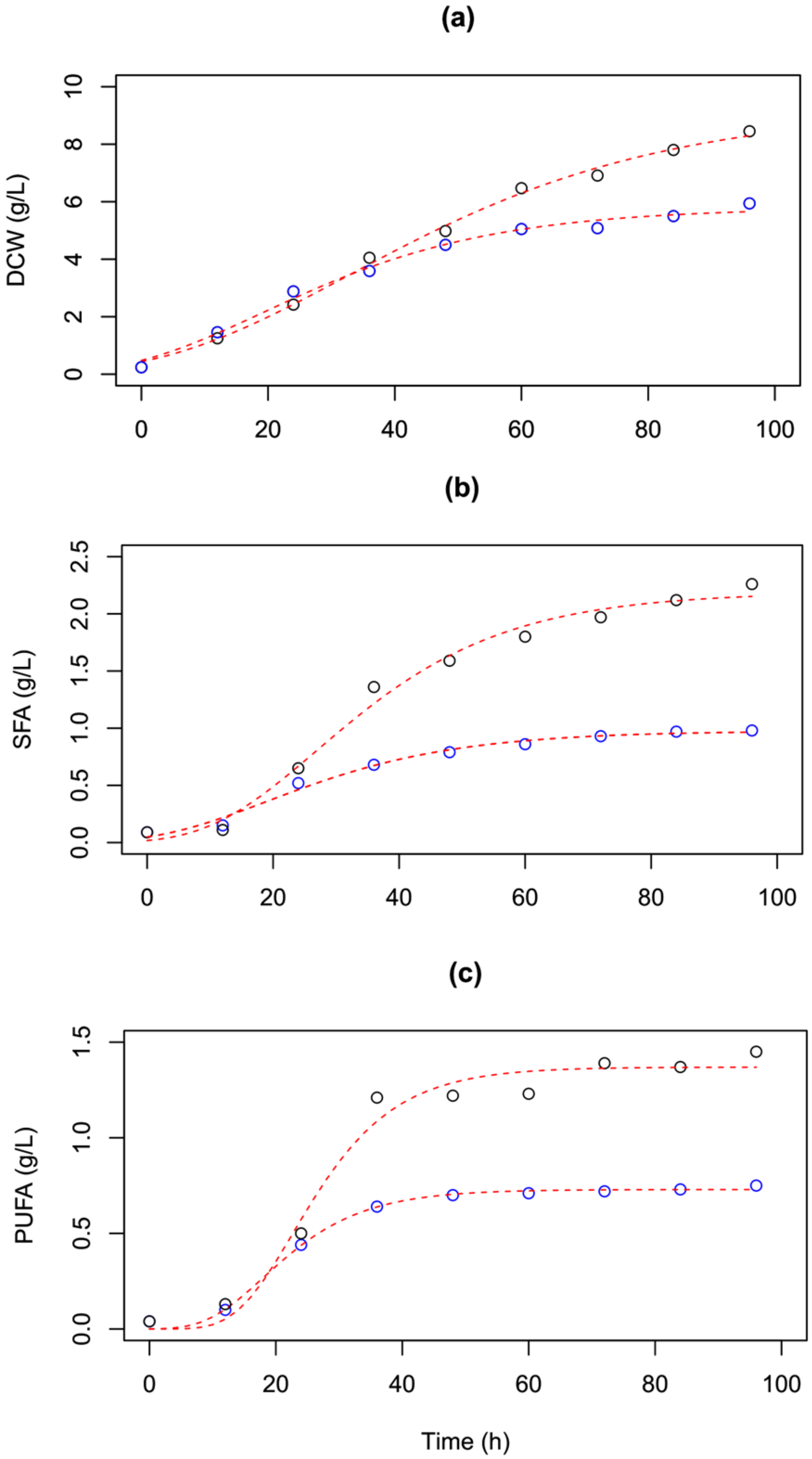

Figure S2. Experimental data and prediction using modified Gompertz model. The ‘o’ (black: w/ MA, blue: w/o MA) and the ‘----' indicate the experimental and predicted data, respectively.

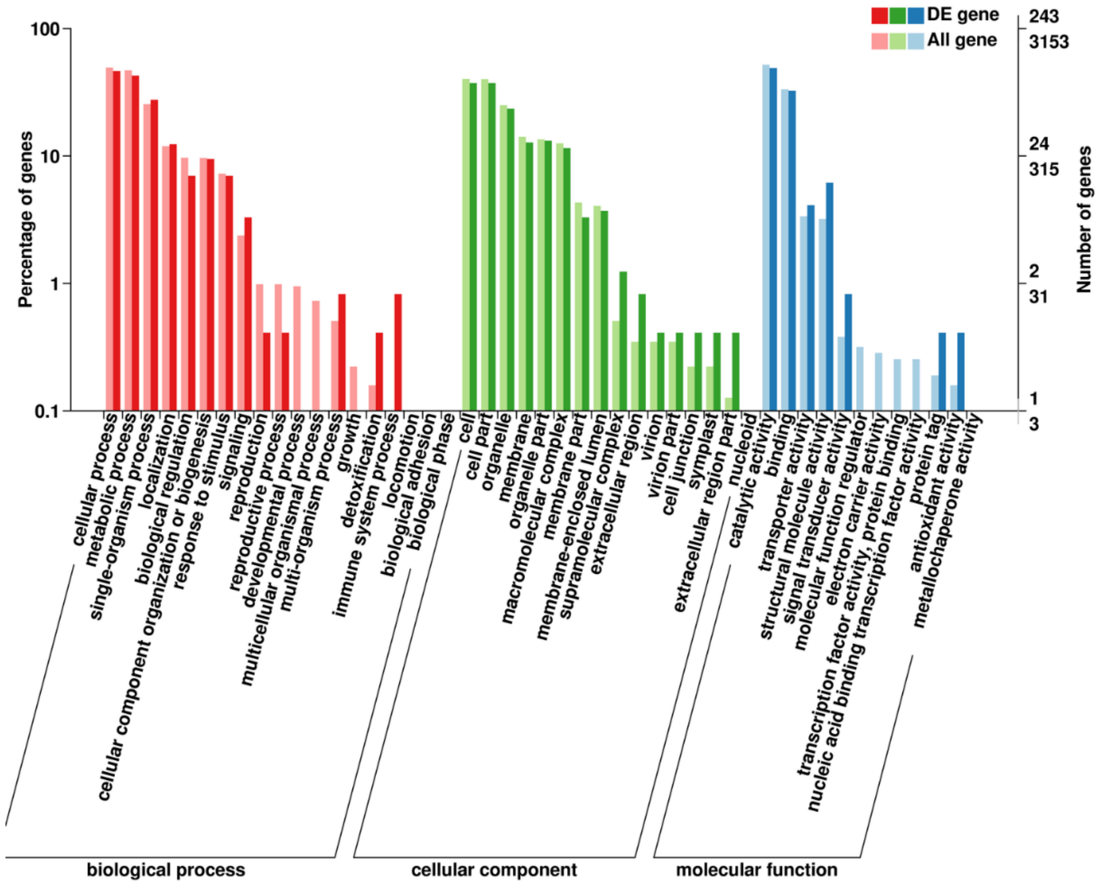

Figure S3. GO classification of differentially expressed genes. The lower and upper numbers on the right y-axis indicate the numbers of total genes and the DEGs, respectively.
